# A Geometric-Feature-Based Method for Automatic Extraction of Anchor Rod Points from Dense Point Cloud

**DOI:** 10.3390/s22239289

**Published:** 2022-11-29

**Authors:** Siyuan Li, Dongjie Yue, Dehua Zheng, Dongjian Cai, Chuang Hu

**Affiliations:** 1 School of Earth Sciences and Engineering, Hohai University, Nanjing 211100, China; 2 Jiangsu Provincial Key Laboratory of Environmental Science and Engineering, Suzhou University of Science and Technology, Suzhou 215009, China

**Keywords:** anchor rod extraction, geometric features, 3D laser scanning

## Abstract

As the technology of high-precision 3D laser scanning becomes increasingly prevalent in the fields of hydraulic building modeling and deformation monitoring, the quality of point clouds plays an increasingly crucial role in data processing. This paper investigates an automatic extraction method of anchor rod points based on geometric features, which focuses on the influence of anchor rod points and mixed pixels in the data of an underground powerhouse of a pumped storage power station on modeling and deformation monitoring during the construction period. This workflow consists of two steps that can automatically extract anchor rod points from high-density point cloud data. Triangular mesh features in the local neighborhood and the parameters of the anchor rods are used to locate the anchor rod in downsampled data, and curvature features are used to extract anchor rod points precisely. The experiment of extracting anchor rods shows that the accuracy of this method of initial identification is 97.2%. Furthermore, precise extraction based on curvature curve fitting is applicable. This method can accurately separate the three types of anchor rods from the dense point cloud on the rough surface of a cavern roof; the false-extraction rate of anchor rod points is about 0.11% to 5.09%. This method can provide high-quality and dependable data sources for the precise registration, modeling and deformation analysis of point clouds in a construction cavern.

## 1. Introduction

In recent years, 3D laser scanning technology has been widely used to create three-dimensional (3D) models and conduct deformation monitoring of underground buildings [1,2,3,4]. Rock reinforcement is a vital link in the process of underground engineering construction. Anchor rods have been widely used in underground engineering and achieve good economic benefits due to their convenient processing, low cost, convenient installation, and high construction efficiency [5,6]. However, during the modeling process, the anchor rods produce sharp meshes on the top of the rods, that damage the esthetics and integrity of the surface model. To obtain the degree of deformation of buildings using high-precision point cloud data, it is necessary to match the multiphase data to the same coordinate frame, and compare the different changes of the multi-phase point cloud data at a specific position [7]. The location of the laser-scanning station varies with the engineering conditions of different data-acquisition periods. Mixed pixels of different directions and magnitudes arise at the rods because of different incident angles during data collection [8]. These undesirable points not only reduce the accuracy of multiphase data registration but also affect the deformation analysis. The extraction of anchor rod points in the raw data enables us to improve the accuracy and efficiency of modeling and eliminate the erroneous results in deformation extraction and analysis.

The raw data of a 3D laser scanner is a jumbled set of 3D coordinates without semantic knowledge of the measurement object [9]. Acquiring specific structural targets from cluttered point cloud data has been a popular research topic in the field of measurement, and rods have received significant attention as one of the common targets in realistic scenarios. The point-based approach is the most common method of extracting rod targets from point clouds, as the point is the most fundamental unit for extraction. Using the features of a single point, a specific threshold is selected, or a classifier is trained based on a specific sample, and the threshold or classifier is used to determine whether the point belongs to a rod target. The construction of effective point cloud features plays an important role in extracting the point cloud target due to the positive correlation between the accuracy of point cloud target extraction and feature validity [10]. The main features, such as reflection intensity-based features [11], geometric morphological features [12,13,14], and descriptor-based features [15,16,17,18], are used to describe point primitives. Reflectance-intensity-based features mainly include the reflection intensity, echo type, and spectral features recorded by laser-scanning systems. The uniqueness of such features depends heavily on the quality of the signal. Geometric morphological features are usually calculated based on local adjacent point sets, including height-difference-based features, eigenvalue-based features, and projection-based features. In addition, geometric morphological features are commonly used to determine the distance from a point to a specific structure (e.g., plane, line), the angle, and the slope between points. Descriptor-based features typically represent local domain point sets with histograms, rotated images, and shape distributions, and feature descriptors are a type of numerical information that cannot be directly understood.

With the rise in artificial intelligence, many feature classifiers, such as Support Vector Machine [19], Random Forest [20], and Joint Boost [11], are also used in point cloud target extraction. Martínez-Sánchez et al. [21] trained a deep network consisting of two autoencoders and an output layer for detecting anchor rods in slopes. In their study, the anchor rods were used as the basis for data alignment, so they were more concerned with finding more anchor rods rather than the identification accuracy. Singh et al. [22] chose an artificial neural network to extract rock bolts in underground coal mines using local point descriptors such as the proportion of variance (POV), radial surface descriptors (RSD), and fast point feature histograms (FPFH). Their algorithm could only recognize a circular region over bearing plates instead of a square and determine whether a bolt exists based on the detected region. Subsequently, Singh et al. [23] proposed a more robust workflow using a multiscale Canupo classifier and a random sample consensus (RANSAC) shape-detection algorithm to distinguish roof bolts from other structures in a mine using the geometric properties of the roof bolt in the 3D point cloud. Gallwey et al. [24] combined machine learning with domain attribute-based feature descriptors and constructed a 65-dimensional feature vector for each point, and used the density-based spatial clustering of applications with noise (DBSCAN) algorithm to divide the results into candidate bolt objects. Saydam et al. [25] presented a practical algorithm, CFBolt, to detect rock bolts from a 3D laser-scanned point cloud. It computed a single-scale proportion of variance (POV) for each point as the local point descriptor and filtered out near 95% of the not-bolt points with a simple but effective classifier, Linear Discriminant Analysis (LDA). They built Point-Net-based rock bolt detection neural network (BRNN) for the accurate detection of anchor rod structures.

The main difference between deep learning and traditional pattern-recognition methods is that deep learning automatically learns features from big data instead of using hand-designed features. Deep learning can quickly find new and effective features from training data for new applications and automatically learn features that can contain thousands of parameters [26]. Although deep learning is intensively studied and partially applied in image analysis and understanding, it is just emerging in point cloud analysis. Meanwhile, point cloud filtering, segmentation, and feature extraction are time-consuming. This leads to low computational efficiency, and related high-performance computing research is still lacking. Deep-learning-based target-recognition algorithms do not demonstrate advantages when facing engineering projects with a large amount of data, short cycle time, insufficient computational conditions, and a simple structure of extracted targets. Thus, this paper introduces a geometric-feature-based underground cavern anchor rod extraction method, which can accurately and effectively identify the anchor rod points in dense point cloud data. First, the extremely dense point cloud data are downsampled to reduce the data volume and thus preserve the contour structure of the anchor rod; in the downsampled data, the local neighborhood detection method of the point cloud is used to extract the suspected anchor rod points, and the suspected anchor rod points are clustered into independent point cloud clusters using distance clustering. Additionally, for each cluster of suspected anchor rod points, the anchor rod points are identified using the threshold method according to the leakage length of the anchor rod. Finally, the adaptive curvature thresholding method is used to accurately extract the anchor rod points of each cluster.

The organization of the paper is as follows. Section 2 presents our method for anchor rod extraction, which includes preliminary extraction and the identification of suspected anchor rod structures, as well as the exact extraction method for anchor rod points. Section 3 discusses the dataset and experimental results used to test our method. Finally, Section 4 describes the outlook and provides a conclusion.

## 2. Methods

**Figure 1:**
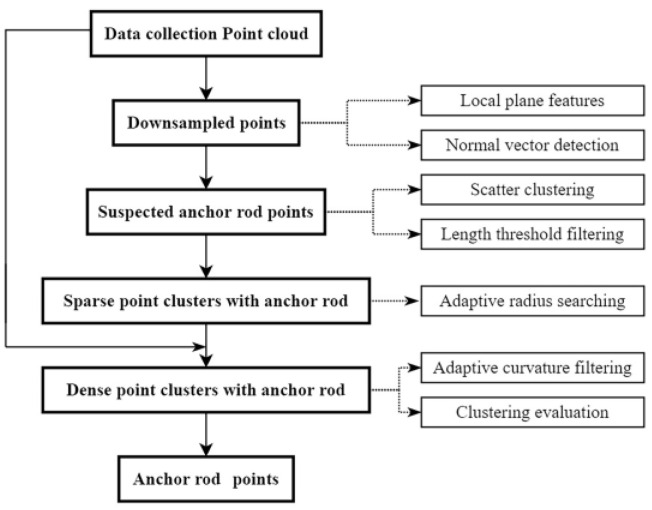
General workflow.

After pouring concrete on the surface of the cavern, the surface around the anchor rods is uneven, the surrounding surface has different undulation states, and the anchor rods are relatively sparse and uniformly distributed without mutual shading in the underground cavern. Because of the influence of its exposed length and scanning view, the raw point cloud of anchor rods has mixed pixels. To reduce the influence of the convex concrete surface surrounding the anchor rods on the accuracy of anchor point extraction, two steps of preliminary identification and accurate extraction of anchor rod points are designed to extract the anchor rod points of the cavern surface support with precision. The purpose of the initial extraction is to detect the location of the anchor rods and extract a local point cloud from the raw data that contain the anchor rods; then, by exact extraction, the anchor rod points are obtained. Figure 1 depicts the adaptive extraction process of anchor rod points based on the preceding procedure.

### 2.1. Preliminary Identification of Anchor Rod Points

#### 2.1.1. Downsampling and Local Coordinate Frame Transformation

The raw point cloud must be uniformly downsampled to improve the initial identification efficiency while retaining the anchor rod characteristics. The point spacing parameter of the downsampling must be determined based on the size of the anchor rod and must meet the requirement to maintain intact anchor rod shape after sampling.

Let $p{\left(x,y,z\right)}^{T}$ be a point in the downsampled point cloud; all neighbor points $P_{i}{\left(x_{i},y_{i},z_{i}\right)}^{T}$ (*i* = 1, 2, …, *n*) are obtained by **p** as center and *R* as radius, where *n* is the number of points in **P**. The selection of the neighborhood radius *R* is dependent on the actual length of the exposed anchor rods; typically half the average exposed anchor length.

The coordinates of the center of gravity **m** of **P** are determined as follows:

(1) $$m=\frac{1}{n}\sum_{i=1}^{n}P_{i}$$

and the covariance matrix of **P** is given by:

(2) $$C=\frac{1}{n}\sum_{i=1}^{n}P_{i}P_{i}^{T}-mm^{T}$$

The matrix of eigenvectors can be calculated by performing Principal Component Analysis on the covariance matrix **C**, as follows:

(3) CU=AU

where **U** is the matrix of eigenvectors, and **A** is the diagonal matrix of the eigenvalues of **C**. The point cloud **P** can be transformed to the local barycentric coordinate by applying Hotelling transform [27]. Let $P^{\prime }$ represent the transformed point cloud, which is calculated by:

(4) $$P^{\prime }=U(P-m)$$

#### 2.1.2. Triangular Mesh Normal Vector and Coordinate Threshold Screening

Delaunay Triangulation [28] is used to build triangular meshes on the two-dimensional plane points obtained by projecting $P^{\prime }$ onto the *XOY* plane. These 2D triangular meshes can be upgraded to 3D triangular meshes by adding corresponding Z coordinates to the projected points. Denote each 3D triangular mesh as $Tr_{j}{\left(x_{l},y_{l},z_{l}\right)}^{T}$ (*l* = 1, 2, 3), where *j* represents the number of meshes.

The normal vector $e_{j}$ of each triangular mesh can easily be calculated from the equation of the plane in which the mesh lies [29].

(5) $$e_{j}Tr_{j}=1$$

The angle $\alpha_{j}$ between $e_{j}$ and the ***Z***-axis is given by:

(6) $$\theta_{j}=\arccos\frac{e_{j}\cdot Z}{\left|e_{j}\right|\cdot \left|Z\right|}$$

(7) $$\alpha_{j}=\left\{\begin{array}{ll} \theta_{j} & ,if\;\theta_{j}\leq 90^{\circ }\\ \theta_{j}-90^{\circ } & ,else \end{array}\right.$$

A threshold β is given to preliminary filter the points; if $\alpha_{j}>\beta$, it is suspected that at least one of the three vertices of the triangular mesh $Tr_{j}$ belongs to the anchor rod point.

Since the surface around the anchor rod is not a regular plane under normal conditions, and often contains irregularly staggered tiny bumps of varying sizes, and the degree of concave and convex undulation is much smaller than the convex form of the anchor rod, the points suspected of belonging to the anchor rod may belong to the concave or convex portion of the surface of $P^{\prime }$ and be determined by screening the threshold β. The three vertices in the triangular mesh are further filtered by setting the threshold *Dist* based on the fact that the length of the bare portion of the anchor rod is significantly greater than the height of the projection and the depth of the depression on the surface of the top arch. When the absolute value of *Z* coordinate of a triangular mesh vertex is greater than *Dist*, that vertex is identified as a possible anchor rod point.

#### 2.1.3. Clustering and Discrimination of Suspected Anchor Rod Points

The initial identified suspected anchor rod points show block distribution characteristics in space. To facilitate the subsequent acceptable identification, the distance clustering method is used to gather these block-distributed points to obtain the suspected anchor rod point cluster. The clustering distance parameter is determined by the initial identification downsampling parameter. Given that the spacing between each support anchor is much greater than the point spacing after downsampling, the distance parameter is set to twice the set point spacing during downsampling to collect the suspected anchor rod points of block distribution into independent suspected anchor rod point clusters $M_{k}$, where *k* is the number of point clusters.

Since complete anchor rod points cannot be obtained during the initial identification process, point cloud clusters containing anchor rod points are extracted from the raw data using the center of gravity of each cluster and the length of the main direction as the initial radius. Subsequently, the acquisition radius is increased in increments of 0.1cm until the range of the *Z* coordinate in the local coordinate frame of the dense point cloud cluster acquired from the raw point cloud does not change. From the raw data, a dense set of point clouds $Y_{k}$ with suspect anchor rod points is obtained.

Hotelling transform is used to transform the point cloud $Y_{k}$ to the local coordinate frame, and ${Y_{k}}^{\prime }$ represents the transformed point cloud. After the transformation, the points belonging to the top arch part are located close to the *XOY* plane, and the main direction of the points belonging to the anchor rod part is nearly parallel to the *Z* axis.

Calculate the maximum distance from the point in ${Y_{k}}^{\prime }$ to the XOY plane, which is defined as $dist_{k}$ and determine if the original set $Y_{k}$ of suspected anchor rod point cloud contains anchor rod points based on the minimum length of the exposed anchor rod and $dist_{k}$.

### 2.2. Precise Extraction of Anchor Rod Points

#### 2.2.1. Curvature Estimation

To resolve the problem of low curvature caused by the mixed pixel phenomenon at the top of anchor rods during the data-acquisition process, the points at the top of anchor rod are extracted according to the $z_{i}$ (*i* = 1, 2, …,$n_{Yk}$) in the local coordinate frame before the curvature is calculated, where $n_{Yi}$ is the number of points in the point cloud in which anchor bolt points exist. The threshold $Z_{\lim}$ is calculated using the following equation:

(8) $$Z_{\lim}=\frac{1}{2}\max(\left|z_{i}\right|)$$

The point satisfying $\left|z_{i}\right|>Z_{\lim}$ is the top point of the anchor rod.

The curvature of the remaining points needs to be calculated. Let q(x,y,z) be one of the remaining points; calculate the eigenvalue matrix of the local neighborhood point cloud centered on point *q* with radius *r* using Equations (1)–(3), and determine the curvature $Q_{q}$ of point *q* using three eigenvalues, $\lambda_{1}$, $\lambda_{2}$, and $\lambda_{3}$ [30,31].

(9) $$Q_{q}=\frac{\min(\lambda_{1},\lambda_{2},\lambda_{3})}{\lambda_{1}+\lambda_{2}+\lambda_{3}}$$

To avoid inaccurate extraction due to a low curvature value of the anchor rod points caused by a neighborhood radius value *r* that is too small, the radius value *r* must be greater than the anchor rod diameter.

#### 2.2.2. Curvature Threshold Determination

After obtaining the curvatures of all points in each point cloud, the curvature values of each point are ordered from smallest to largest, and the Gaussian function model is applied to fit the ordered curvatures.

(10) $$y=a_{1}e^{-{\left(\frac{x-b_{1}}{c_{1}}\right)}^{2}}+a_{2}e^{-{\left(\frac{x-b_{2}}{c_{2}}\right)}^{2}}+a_{3}e^{-{\left(\frac{x-b_{3}}{c_{3}}\right)}^{2}}$$

where *x* represents the serial number of the point and *y* represents the curvature of the point; $a_{1}$, $b_{1}$, $c_{1}$, $a_{2}$, $b_{2}$, $c_{2}$, $a_{3}$, $b_{3}$, and $c_{3}$ are the parameters to be fitted. The maximum curvature point of the fitted curve is computed, and the corresponding point’s curvature value is used as the curvature threshold *T* of this point cloud to differentiate the anchor rod points from the surrounding surface.

After filtering through the curvature threshold, some top arch points with relatively large concavity and convexity may be considered anchor rod points, which are usually distributed in blocks; these points are clustered by distance. After clustering, when the difference between the maximum and minimum values of the *Z* coordinate of each block of the point cloud is greater than *Dist*, they are regarded as anchor rod points; otherwise, they are considered non-anchor rod points.

#### 2.2.3. Evaluation of Extraction Results

After extracting the anchor rod points using the method described above, the extraction accuracy is assessed by defining the false-extraction rate *F*, which is defined as follows:

(11) $$F=\frac{n_{3}-n_{2}}{n_{1}}$$

where $n_{1}$ represents the number of points in the cluster, $n_{2}$ represents the number of anchor rod points extracted by manual discrimination, and $n_{3}$ represents the number of points extracted by this method. The lower the final false-extraction rate *F*, the greater the extraction precision of anchor points.

## 3. Experiment and Results

### 3.1. Experimental Data

The anchor rod support method is an essential method used in underground engineering and all types of underground projects to improve stability and maintain a certain bearing capacity during construction. To ensure the safety of construction during the excavation phase of an underground plant of a pumped storage power station, deformation caused by geological conditions, blasting, and other factors must be monitored regularly.

**Figure 2:**
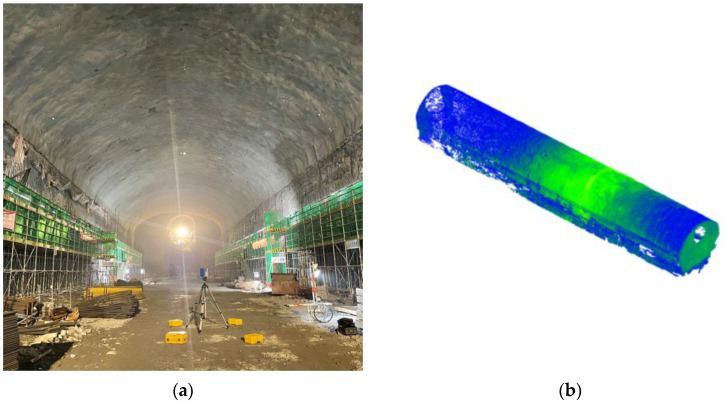
Experimental data acquisition. (**a**) Data acquisition site. (**b**) Raw point cloud.

This paper’s experimental subject is a cavern room during the construction of the main underground plant of a pumped storage power station in Zhejiang Province (see Figure 2a). The experimental data acquisition was performed by scanning the cavern room with a Z+F IMAGER 5016 3D laser scanner with a scan resolution of 0.6 mm (10 m), and the collected dense point cloud data on the surface of the cavern room are shown in Figure 2b.

**Figure 3:**
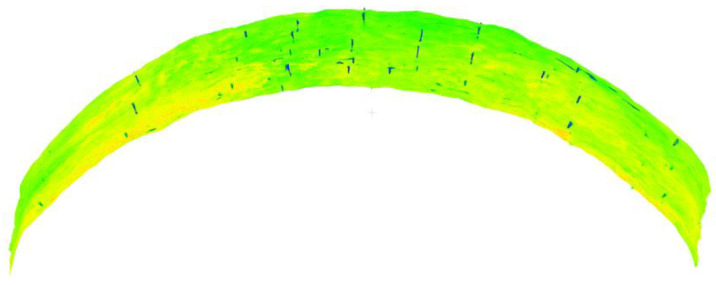
Experimental dataset.

During the construction period, the cavern roof had an uneven distribution of anchor rods and different exposed lengths, and the same anchor rod point cloud obtained from different stations was not exactly the same due to mixed pixels, which had a significant impact on the accuracy of cavern point cloud alignment and deformation analysis. Therefore, the anchor rod point adaptive extraction method based on geometric features proposed in this paper was used to extract anchor rod points automatically. The test area was a 12 m-long cavern roof point cloud, and the diameter of the roof support anchor rod was about 4 cm; the test area is shown in Figure 3.

### 3.2. Preliminary Identification

**Figure 4:**
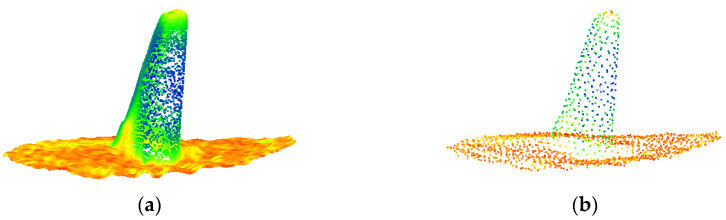
Point cloud containing anchor points. (**a**) Raw point cloud. (**b**) Downsampled point cloud.

This region’s point cloud was downsampled uniformly to produce a point cloud with a 1 cm point spacing. Figure 4a depicts the local point cloud of a particular anchor rod structure, and Figure 4b shows that the point cloud retains the original structure characteristics of the anchor rod after downsampling.

**Figure 5:**
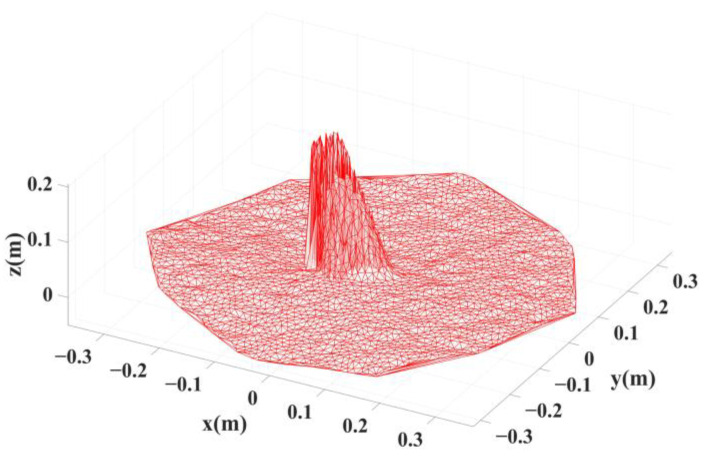
Triangular meshes in a 3D coordinate frame.

All points are first projected onto the *XOY* plane in the local coordinate frame, and two-dimensional triangular meshes are constructed through these projected points. Subsequently, the triangular mesh vertices in the two-dimensional plane are assigned the original *Z* coordinate values, and the triangular meshes in the three-dimensional coordinate frame are formed, as shown in Figure 5.

**Figure 6:**
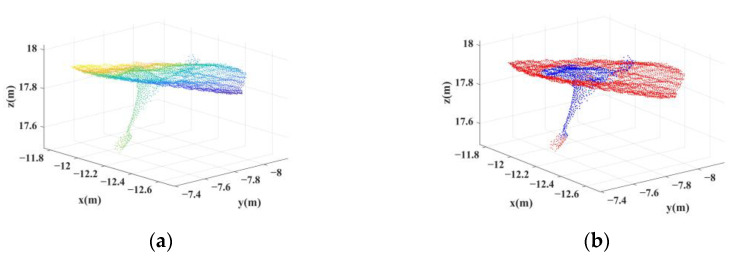
A neighborhood containing anchor rod points. (**a**) Initial point cloud after downsampling. (**b**) Preliminary identification results; points colored in blue are suspected anchor rod points.

**Figure 7:**
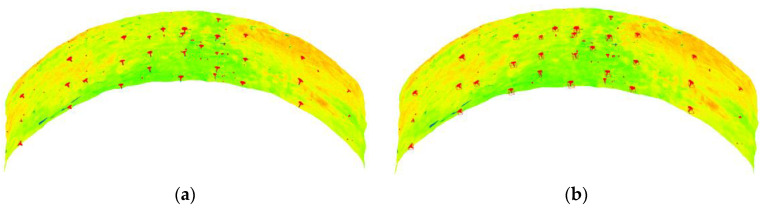
Suspected anchor rod points of initial identification. (**a**) Unclustered suspected anchor rod points in the test area. (**b**) Clustering results of suspected anchor rod points.

By setting the angle threshold β=60° and according to the minimum length *D* = 0.1 m of exposed anchor rods in the actual condition, the suspected anchor points were extracted using the initial identification method of anchor rods proposed in this paper from the downsampled point cloud. Among them, the downsampled point cloud in the neighborhood of a point of the anchor rods is shown in Figure 6a, and the suspected anchor rod points obtained after the initial identification are shown in Figure 6b. All suspected anchor rod points were obtained by the initial identification of the point in the roof of the 12 m cavern, which is shown in Figure 7a. The extracted suspected anchor rod points were clustered by distance, and a total of 165 clusters of the suspected anchor point cloud were obtained (see Figure 7b), including the anchor rod structure and the tiny misidentified roof projection.

**Figure 8:**
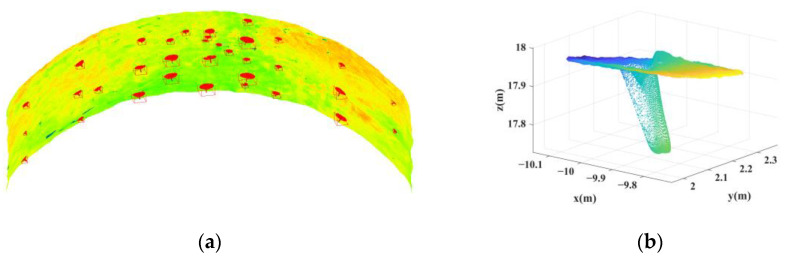
Precise screening results after clustering. (**a**) Clusters with anchor rod points. (**b**) A cluster containing anchor rod points.

We can determine whether there is an anchor rod point in the cluster of point clouds based on the minimum exposed length of the anchor rod and the maximum absolute value of the *Z* coordinate value of each cluster of point clouds in the local barycenter coordinate frame. As shown in Figure 8a, after discrimination, 34 of the 165 initial point cloud clusters suspected to contain anchor rod points were found to contain anchor rod points. We can obtain dense point clusters from the raw data using the barycenter of each cluster as the center and the length of the cluster’s main direction as the radius. Then, we can increase the search radius by 1 cm increments until the range of *Z* coordinate values of the acquired point cluster under the local center of gravity coordinate frame no longer varies. Thus, we obtain dense point clusters containing anchor rod points, as shown in Figure 8b.

In this test area, 34 sets of anchor rod point clouds were obtained. To validate the results of automatic identification, manual discrimination was used to manually count the number of real anchor rods in the test area. The test area contained 36 anchor rods, 2 of which were located in the same point cluster due to their close proximity, and 1 anchor rod was not detected because its exposed length was significantly shorter than the exposed lengths of the others due to blasting and other construction-related factors. The method described in this paper is comparable to the number of manually identified anchor rods, accurately identifies normal exposed supporting anchor rods, and has an initial identification accuracy of 97.2% in this experimental area.

### 3.3. Precise Extraction

**Figure 9:**
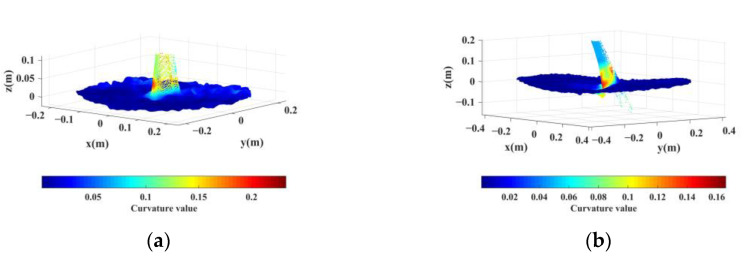
Curvature distribution of clusters containing anchor points. (**a**) Anchor rod in normal condition. (**b**) Anchor rod with significantly mixed pixels.

For each of the 34 point clouds with a confirmed anchor rod in the initial identification, the point cloud at the top of the anchor rod is extracted using the threshold value $Z_{\lim}$ in Equation (8). The radius *r* of the neighborhood is set to 5 cm based on the diameter of the anchor rod, and the curvature of points in each cluster is computed using Equation (9). Figure 9 depicts the curvature distribution with two types of anchor rods.

Figure 9 demonstrates that the curvature of the top arch portion and the anchor rod portion in different point clusters are distinct and can be distinguished based on their respective curvatures. The curvature data are fitted using Equation (10), the curvature threshold of each point cloud cluster is computed using the fitting curve, and the anchor rod points in 34 groups of point cloud clusters are extracted precisely. Table 1 contains the counts of the points in each cluster $n_{1}$, the number of points extracted by manual discrimination $n_{2}$, the number of points extracted by this method $n_{3}$, and the false-recognition rate *F* for each point cluster.

According to Table 1, the number of anchor rod points extracted by the method described in this paper is slightly greater than the manually extracted ones, and the misidentification rate ranges from 0.11% to 5.09%. Most misidentified points are located at the intersection of the anchor rod and the top arch; this is the transition area between the anchor rod points and the top arch points. Consequently, there is an over-identification phenomenon.

**Figure 10:**
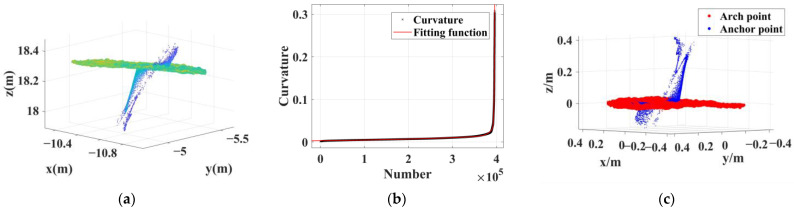
Extraction results of long anchor rod with significantly mixed pixels. (**a**) Dense point cloud. (**b**) Curvature fitting results. (**c**) Extraction results; red points belong to the arch roof, and the blue points belong to the anchor rod.

**Figure 11:**
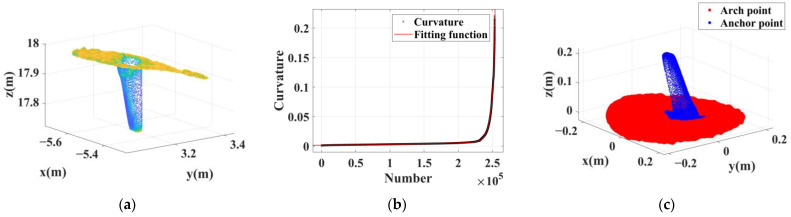
Extraction results of short anchor rod with a few mixed pixels. (**a**) Dense point cloud. (**b**) Curvature fitting results. (**c**) Extraction results; red points belong to the arch roof, and the blue points belong to the anchor rod.

**Figure 12:**
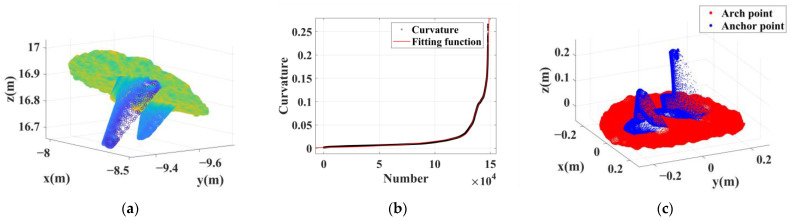
Extraction results of two anchor rods in the same cluster. (**a**) Dense point cloud. (**b**) Curvature fitting results. (**c**) Extraction results; red points belong to the arch roof, and the blue points belong to the anchor rod.

The 34 sets of anchor rod point clouds extracted from this test area can be divided into 3 types based on their characteristics. Type 1 is a cluster of point clouds containing long anchor rods, totaling nine groups. Such anchor rod point clouds mostly have significantly mixed pixels, and most of them have reverse trailing points, which are characterized as anchor rod point cloud No.1, as shown in Figure 10. Type 2 contains anchor rod point clouds with a small number of mixed pixels, 24 groups in total; the length of such anchor rods is about 15–20 cm, and there are a small number of mixed pixels on the back of the scanned line of sight, Furthermore, the anchor rod morphology of the point cloud is well maintained; its characteristics are shown in Figure 11 for anchor rod point cloud No.13. Type 3 is a cluster of multianchor rod-aggregation-type point clouds, with only one group, which is characterized as No.7 (see Figure 12).

According to the findings above, when the anchor rods are not perpendicular to the top arch surface, there is a phenomenon known as over-identification. This phenomenon is primarily found in orientations where the angle between the anchor rods and the top arch surface is less than 90°. It is almost nonexistent in orientations where the angle is greater than 90°. This method can accurately extract anchor rod points from shotcrete surfaces of an arch roof with various feature types and sparse distribution. The number of manually extracted anchor rod points is less than the number extracted by this method, but this method still meets the needs of subsequent data application processing.

## 4. Conclusions

This paper suggests an adaptive extraction method of anchor rod points based on geometric features to address the issue wherein the anchor rod and mixed pixels on the roof of the underground cavern affect the accuracy of point cloud modeling, precision registration, and deformation analysis. The designed anchor rod points’ initial identification and fine extraction steps can accurately extract anchor rod points from the dense point cloud of an irregular shotcrete roof arch. The suspected anchor rod points are initially screened using the normal vectors of the mesh and *Z*-coordinate features under the local coordinate frame. All anchor rod point clusters are obtained using the distance clustering method. The accuracy of the number of anchor rods after initial identification and clustering is experimentally verified to be 97.2% based on the actual exposed length of anchor rods. An anchor rod with an externally exposed length of more than 10 cm is always recognized. By downsampling the initial dense point cloud data in advance and keeping the edge features of anchor rods, it is possible to significantly increase the extraction efficiency of support anchor point clouds. The proposed geometric-feature-based adaptive-extraction method has good applicability and stability. The error-recognition rate of the arch point ranges from 0.11% to 5.09%. This method can provide high-quality data for accurate registration, deformation monitoring, precise modeling, and other data processing of three-dimensional laser scanning point clouds for hydropower construction caverns, high-speed railway tunnels, high slopes, and other anchor rod support projects without the influence of anchor rod points.

## Figures and Tables

**Table 1:** Extraction results of anchor rod points in each cluster.

No.	$n_{1}$	$n_{2}$	$n_{3}$	*F*	No.	$n_{1}$	$n_{2}$	$n_{3}$	*F*
1#	247,624	9667	11,644	0.80%	18#	35,809	5003	5341	0.94%
2#	396,670	5367	8794	0.86%	19#	256,555	16,371	23,754	2.88%
3#	78,174	11,174	14,497	4.25%	20#	257,904	17,634	21,362	1.45%
4#	134,365	9115	14,365	3.91%	21#	286,312	4953	7213	0.79%
5#	158,955	4285	5427	0.72%	22#	698,531	12,314	15,644	0.48%
6#	512,638	15,680	16,726	0.20%	23#	722,836	24,422	28,517	0.57%
7#	150,623	16,373	22,566	4.11%	24#	369,082	12,908	19,305	1.73%
8#	217,695	10,617	19,502	4.08%	25#	335,320	14,253	16,160	0.57%
9#	641,686	8433	13,106	0.73%	26#	746,623	6532	7337	0.11%
10#	373,780	12,056	20,255	2.19%	27#	343,619	10,365	12,616	0.66%
11#	110,280	5346	9556	3.82%	28#	165,935	6465	7312	0.51%
12#	96,804	4885	6693	1.87%	29#	362,603	12,057	15,770	1.02%
13#	175,568	10,639	15,686	2.87%	30#	252,258	9151	21,985	5.09%
14#	149,944	8885	14,344	3.64%	31#	500,517	11,637	19,944	1.66%
15#	146,863	12,105	16,663	3.10%	32#	50,507	3898	4531	1.25%
16#	1,126,246	11,283	12,903	0.14%	33#	67,380	9084	9702	0.92%
17#	192,904	3453	5894	1.27%	34#	377,817	13,361	22,259	2.36%

## Data Availability

Not applicable.
